# Concentrations of lead and other elements in the liver of the white-tailed eagle (*Haliaeetus albicilla*), a European flagship species, wintering in Eastern Poland

**DOI:** 10.1007/s13280-017-0929-3

**Published:** 2017-06-15

**Authors:** Ignacy Kitowski, Dariusz Jakubas, Dariusz Wiącek, Agnieszka Sujak

**Affiliations:** 1 State School of Higher Education in Chełm, Pocztowa 54, 22-100 Chełm, Poland; 2 Department of Vertebrate Ecology and Zoology, University of Gdańsk, Wita Stwosza 59, 80-308 Gdańsk, Poland; 3 Institute of Agrophysics, Polish Academy of Sciences, Doświadczalna 4, 20-290 Lublin, Poland; 4 Department of Biophysics, University of Life Sciences in Lublin, Akademicka 13, 20-933 Lublin, Poland

**Keywords:** Bioaccumulation, Lead ammunition, Liver, Raptors, Trace elements

## Abstract

As a top predator, the white-tailed eagle (*Haliaeetus albicilla*) may serve as a good indicator species, providing information about the bioavailability of contaminants and their transfer within the food chain. In this study, we aimed to determine the common sources of origin of 17 metals and other elements in the liver of white-tailed eagles, and to compare the variations in their hepatic concentrations by age (adults vs immatures) and sex (males vs females) in groups of white-tailed eagles wintering in Eastern Poland. The element concentrations followed the pattern of S > K > Na > Fe > Mg > Ca > Zn > Cu > Mn > Se > Pb > Hg > Cd > Cr > Sr > V > Sc. We found significant age-related differences in the hepatic concentrations of some of the elements. Adults showed higher concentrations of Pb, Cd, Ca, Fe, and Zn and lower concentrations of Cu and Se than immatures. These differences may be explained by age-related differences in wintering strategy (adults are sedentary, and immatures are migratory) and hunting skills (adults are more successful when hunting for agile prey). Our study indicates that ingested Pb ammunition poses a serious threat to the health and lives of white-tailed eagles in Poland (32% of the studied individuals had acute lead poisoning). Our study also indicates a serious need for banning the use of lead hunting ammunition in the parts of Europe (including Poland) where it is still allowed.

## Introduction

As biomonitoring sentinels, raptors can provide early warnings of the potential impacts of contaminants on humans and the environment and can act as a means of tracking the success of associated mitigation measures (Kim et al. 2008; Ansara-Ross et al. 2013; Gomez-Ramírez et al. 2014). The value of birds as biomonitors of environmental pollution has been widely recognized (Horai et al. 2007; Schummer et al. 2011; Kim and Oh 2015). Owing to their top position in food webs, relatively long lifespan over which contaminants are accumulated, and exposure over different temporal and spatial scales (Jager et al. 1996; Castro et al. 2011; Kim and Oh 2015), birds of prey are considered to be good avian sentinels of environmental contamination. Their response to chemicals ranges from residue accumulation to population decline (Gomez-Ramírez et al. 2014).

Livers are frequently used for bioindication purposes (Horai et al. 2007; Castro et al. 2011; Schummer et al. 2011; Kalisinska et al. 2014a). On the one hand, this organ serves as a reservoir of many vital substances; on the other hand, it plays an important role in the detoxification of accumulated toxic material (e.g., toxic heavy metals). Research indicates that proteins rich in sulfur amino acids play an important role in detoxification. Detoxification processes are determined by the proper intake of sulfur (in the form of sulfur amino acids) (Tamas and Martinoia 2006; Nimni et al. 2007; Toohey 2014).

The white-tailed eagle, *Haliaeetus albicilla*, has been especially promoted as a sentinel species for various environmental contaminants. It is a long-lived top predator with a wide home range. This species may perform partial migration (Cramp and Simmons 1980; Nadjafzadeh et al. 2016a). As eagles are often considered umbrella, flagship, and keystone species, they are of high conservation priority, and their health state (i.e., contamination level) is important to assess the population condition. Currently, the white-tailed eagle population in Europe is undergoing a considerable increase and territorial expansion due to the cessation of persecution and the massive use of pesticides (especially DDT), as well as the effective protection of nesting sites (Deinet et al. 2013). In Poland, this species is widely distributed. Its population is estimated to be 1400–1500 breeding pairs (Chodkiewicz et al. 2013, 2015), making up 11% of European population and constituting the largest European mainland breeding population (Deinet et al. 2013). However, the Polish population is less genetically diverse than populations in other Central and East European countries. This lower diversification may have resulted from a bottleneck in the twentieth century (Hailer et al. 2007; Langguth et al. 2013). The white-tailed eagle is associated with aquatic habitats. It breeds in Central and Eastern Europe in coastal areas and wide river valleys that are rich in lakes and fish ponds (Cramp and Simmons 1980; Nadjafzadeh et al. 2016a). Females (4–6 kg) are slightly heavier and larger than males (3.5–4.5 kg) (Cramp and Simmons 1980; Mizera 2004), and a breeding pair occupies a hunting territory of 100 km^2^. The diet of the white-tailed eagle mainly consists of fish and waterbirds (Cramp and Simmons 1980; Mizera 2004; Nadjafzadeh et al. 2016a). It hunts for 0.100–8.0-kg fish, preferring a 0.5–3.0 kg range. In Poland, the species mainly preys on crucian carp, *Carassius carassius* (at fish farms); bream, *Abramis brama*; northern pike, *Esox lucius*; and roach, *Rutilus rutilus* (in lakes) (Zawadzka 1999, Zawadzka et al. 2006). Coots, *Fulica atra*, and mallards, *Anas platyrhynchos*, are the most common avian prey (Zawadzka 1999; Zawadzka et al. 2006), and European white-tailed eagles, especially immature individuals in winter, regularly forage on carrion (Zawadzka 1999; Mizera 2004; Zawadzka et al. 2006). In Central Europe, including Poland, adults are sedentary and winter in their territories, while immatures migrate up to a few hundred km. (Cramp and Simmons 1980; Mizera 2004; Nadjafzadeh et al. 2016a).

The aims of this study were to (1) determine the hepatic concentrations of metals and elements in 22 white-tailed eagles from Eastern Poland, (2) compare the variations in the hepatic concentrations of 17 elements by age (adults, immatures) and sex (males, females), and (3) compare the concentrations of elements between birds collected in two regions (southern and northern) of Eastern Poland that differ in their habitat composition (Table 1).

**Table 1:** Relative area (%) of particular habitats in 20-km buffers around the sampling sites [according to the CORINE Land Cover model (CLC2012)] in the northern (N, *N* = 13) and southern (S, *N* = 7) regions of the study area

Region	Arable land	Pastures	Artificial surfaces	Forests	Water bodies	Wetlands
N	38.5	7.1	2.4	36.6	7.3	0.8
S	40.6	9.6	3.4	34.0	1.2	0.8
*χ* ^2^ test	4.42	37.69	15.32	7.38	353.62	0.01
*P*	0.03	<0.0001	0.0001	0.007	<0.0001	0.93

Based on the diet composition of the study species, we expect the main sources of trace element assimilation to be as follows: hunting ammunition (Pb), fertilizers (Cd, Cr, and V), and aquatic prey (Cu, Mn, and Hg). It must be emphasized that Hg, Cd, and Pb are extremely toxic elements. Considering the age-related differences in hunting effectiveness, we expect that immature eagles may hunt in suboptimal areas and focus on suboptimal prey (e.g., carrion) and thus be more prone to Pb contamination than experienced adults. Because sexual dimorphism in raptors affects prey selection (Krüger 2005; Slagsvold and Sonerud 2007), we expect there to be differences between males and females in their exposure to contamination. As white-tailed eagles may exploit prey and carrion contaminated with lead from hunting ammunition (Helander et al. 2009; Nadjafzadeh et al. 2013), we expect elevated lead concentrations, at least in some individuals, which may be associated with the elevated accumulation of other elements. Considering the differences in habitat composition of the regions from which the birds were collected, we may expect higher Pb accumulation in lake lands, where white-tailed eagles may hunt for ducks shot by lead ammunition.

## MATERIALS AND METHODS

### Origin of the studied birds

**Fig. 1:**
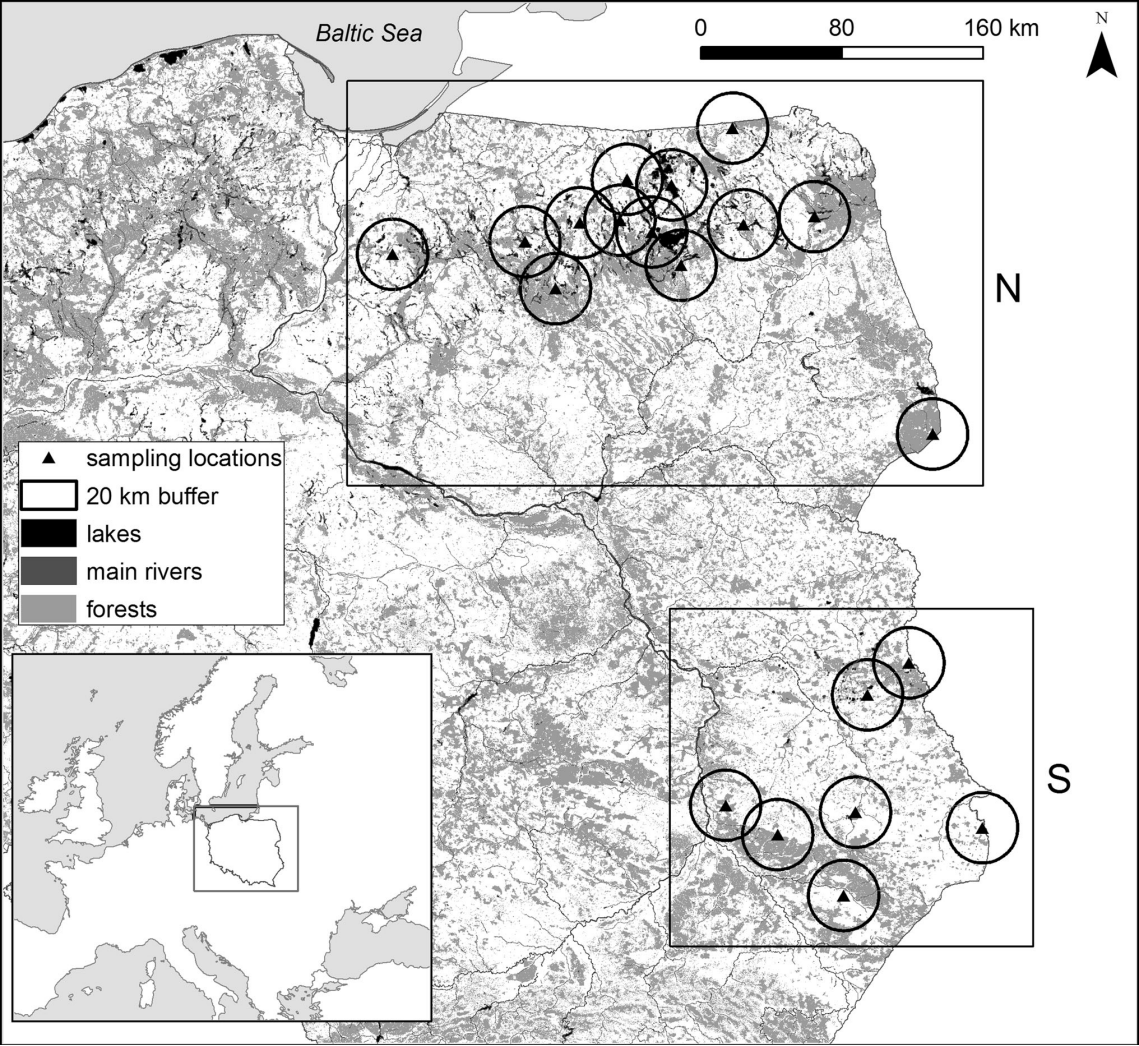
Study area in Eastern Poland divided into northern (N) and southern (S) parts with the locations of sampling sites with 20-km buffers. Two locations are not shown and were not considered for analysis because only two eagles were found in each

Livers were collected and analyzed from 22 dead white-tailed eagles that had been delivered as wounded to veterinary clinics in Eastern Poland (Fig. 1) in winter (December–March) from 2009 to 2014. The birds either died or, because they were untreatable upon delivery, were euthanized by lethal injection by veterinary doctors to spare them from suffering; the total stay of the raptors in the clinics or rehabilitation centers did not exceed 7 days. The birds were sexed by internal examination after dissection and classified as being immature (less than 2 years old) or adult (over 2 years old) on the basis of plumage, gonad development, and iris color (Helander et al. 1989, 2009; Forsman 1999). Data provided on a wet weight (ww) basis were converted to dry weight (dw) using a factor of 0.75, following Kalisińska et al. (2006).

The study area was divided into two sections: the northern region (14 sample sites), which is characterized by a high number of lakes, and the southern region (6 sample sites), which is characterized by a farmland landscape with a smaller number of water bodies. The northern region consists of two subprovinces [according to the physico-geographical regionalization of Poland (Kondracki 2002)]: the Eastern Baltic Lakelands (Pojezierze Wschodniobałtyckie) and the Podlasie–Byelorussian Upland (Wysoczyzny Podlasko–Białoruskie). The first subprovince is characterized by a postglacial landscape with a high number and area of lakes (this subprovince includes the Masurian Lakeland) and forests. The second subprovince in the northern region is an upland area with a small number of lakes but a considerable number of river valleys and inland marshes. The southern region mainly includes two subprovinces: Polesie and the Lublin–Lviv Upland. The first subprovince is a flat area with shallow and wide inland marshes and moraine plains, and it is mainly covered by agricultural areas and forests. The second subprovince is an upland area with river valleys, and is one of the most-developed agricultural regions in Poland. In the northern region, water bodies and forests covered a greater relative area in 20-km buffers around the sampling sites (see details about buffers in “Statistical analyses”) compared with the southern region. By contrast, the relative areas of arable land, artificial cover, and pastures around the sampling sites in the southern region were larger than in the northern region (Table 1).

### Laboratory analyses

Livers were extracted from the corpses of the birds with a ceramic knife and stored in polyethylene bags in the freezer at −30 °C prior to the analyses. The samples were then lyophilized and ground in a ceramic mortar. Experimental methods were followed with modifications to those previously published (Kitowski et al. 2014). All glassware and utensils were rinsed with tap water, soaked in an acid bath (5 M HNO_3_) for 24 h, rinsed with demineralized water and dried under a laminar flow hood before use to minimize the risk of any metal contamination. Weighted portions of the samples (500 ± 1 mg) with 10 mL of concentrated HNO_3_ (Sigma Aldrich) were subjected to wet-ashing, and mineralization was carried out using a microwave digestion system with optical temperature and pressure monitoring of each individual sample during acid digestion (Berghof Speedwave) in Teflon vials (DAP 100 type). The mineralization process ran as follows: 15 min from room temperature to 140 °C, 5 min at 140 °C, 5 min from 140 to 170 °C, 15 min at 170 °C, and cooling to room temperature (variable time); the pressure over the entire mineralization process did not exceed 12 bar. After completing the mineralization process, a clear solution of elements was obtained, which was cooled to room temperature, transferred to a 50-mL volumetric flask and filled with demineralized water (ELGA Pure Lab Classic) to the indicated level. Inductively coupled plasma optical emission spectrometry (ICP-OES) with a Thermo Scientific iCAP Series 6500 analyzer equipped with a charge injection device (CID) was used for the identification of elements. The spectrometer was controlled by PC-based iTEVA software, and the following instrumental settings were used: RF generator power of 1150 W, RF generator frequency of 27.12 MHz, coolant gas flow rate of 16 L min^−1^, carrier gas flow rate of 0.65 L min^−1^, auxiliary gas flow rate of 0.4 L min^−1^, max integration time of 15 s, pump rate of 50 rpm, axial viewing configuration, 3 replicates, and a 20-s flush time. We used the following multi-element stock solutions (Inorganic Ventures) as standards:

(A) *Analityk 46* ^63^Cu, ^57^Fe, ^24^Mg, ^31^P, ^39^K, and ^23^Na in 1000 mg L^−1^ of 5% HNO_3_;
(B) *Analityk 47* ^27^Al, ^75^As, ^111^Cd, ^52^Cr, ^208^Pb, ^55^Mn, ^201^Hg, ^60^Ni, ^45^Sc, ^79^Se, ^88^Sr, ^51^V, and ^66^Zn in 100 mg L^−1^ of 10% HNO_3_;
(C) *Analityk 83* ^63^Cu, ^39^K, ^24^Mg, ^23^Na, ^31^P, and ^32^S in 1000 mg L^−1^ of 5% HNO_3_.

Samples (eagle livers) were run simultaneously with a blank (control) sample, and each liver sample was divided into two subsamples and analyzed in duplicate. A certified reference material, TraceCERT periodic table mix 1 for ICP (Fluka Analytical, Sigma Aldrich), was used to control the accuracy of the method under the existing working conditions, and three randomly selected samples were supplied with known amounts of the analytical standard to calculate the recovery percentage. Concentrations of all the examined elements were used as positive controls (Table 2), and the mean recovery percentages of the analyzed elements were calculated according to the following equation:

$$ {\text{Recovery }}[\% ] = ({\text{CE/CS}} \times 100), $$

where CE was the experimental concentration determined from the calibration curve, and CS was the spiked concentration.

**Table 2:** Validation of the analytical method used in this study: linearity (the ability of the method to obtain test results proportional to the concentration of the analyte), limit of detection (LOD), and recoveries for the studied elements

Element	Linearity correlation *r*	LOD (mg L^−1^)	Recovery (%)
Ca	0.9986	0.002	107
Cd	0.9999	0.001	98
Cr	0.9998	0.002	97
Cu	0.9999	0.002	103
Fe	0.9999	0.012	111
Hg	0.9997	0.052	93
K	0.9995	0.120	105
Mg	0.9982	0.005	109
Mn	0.9998	0.002	96
Na	0.9982	0.005	103
Pb	0.9999	0.008	97
S	0.9985	0.004	106
Sc	0.9998	0.002	99
Se	0.9999	0.011	94
Sr	0.9998	0.003	97
V	0.9999	0.003	95
Zn	0.9998	0.010	103

All the obtained concentrations were presented as mg kg^−1^ dw. To compare the results with data from other authors presented in wet weight, a conversion factor of 4.0 was used because the water content of the liver is estimated to be 75% (Kalisińska et al. 2006). Liver Cd levels ≥3 mg kg^−1^ dw were classified as indicative of increased environmental exposure (Burgat 1990; Battaglia et al. 2005). Shore et al. (2011) proposed hepatic Hg concentrations >6.7 mg kg^−1^ dw to be associated with adverse effects on avian reproduction, and Hg values >67.0 mg kg^−1^ dw may result in death for non-marine birds. Hepatic concentrations of Pb ≥ 6 mg kg^−1^ dw were considered diagnostic of elevated exposure to Pb resulting in subclinical toxicity (Pain et al. 1995; Helander et al. 2009), while ≥15 mg kg^−1^ dw is considered diagnostic of Pb poisoning (Franson 1996); acute Pb poisoning has been observed to occur when liver concentrations exceed 30 mg kg^−1^ (Martin et al. 2008). These thresholds are based on published data that relate hepatic Pb concentrations in raptors to evidence Pb poisoning, as well as subclinical effects (Pain et al. 1995; Franson 1996; Martin et al. 2008). Selenium at the level of 4–10 mg kg^−1^ dw is considered the background concentration in avian liver tissue (Ohlendorf 1989). In this study, a hepatic Se concentration >10 mg kg^−1^ dw was taken as exceeding the background level. In the case of zinc, 200 mg kg^−1^ dw was considered the background concentration in liver tissue (Honda et al. 1990). The Hg/Se molar ratio was calculated based on the molar weights of the above elements: 78.96 g mol^−1^ Se and 200.59 g mol^−1^ Hg.

### Statistical analyses

To identify element concentration patterns, i.e., to find groups of elements with high degrees of association/correlation, we performed the following analyses:

1. Spearman rank correlation;
2. Principal component analysis (PCA) to reduce the number of variables and to assign new ones as factors representing groups of elements with significantly correlated concentrations; this analysis was performed on log-transformed data.

Intergroup differences in element concentrations were investigated using two methods:

1. Multivariate (for all elements together) PERMANOVA (non-parametric MANOVA based on the Bray–Curtis measure; Anderson 2001) with fixed factors (age and sex) and their interaction as explanatory variables;
2. The similarity percentage breakdown (SIMPER) procedure to assess the average percentage contribution of individual factors to the dissimilarity between objects in a Bray–Curtis dissimilarity matrix (Clarke 1993);
3. Univariate analysis (for particular elements) using ANOVA and then a post hoc HSD test for unequal N (Sokal and Rohlf 1981) for normal data and PERMANOVA for non-normal data (Anderson 2001).

Separate Spearman correlations were performed to investigate whether the size of the area of a particular habitat near where the studied white-tailed eagles were found (sample site) is related to the hepatic concentrations of particular elements. First, the areas of the six most important habitats, arable land, pastures, artificial surfaces (including urbanized and industrial areas), forests, water bodies, and wetlands, in 20-km buffers around where white-tailed eagles were found and delivered to veterinary clinics were calculated based on the CORINE Land Cover model (CLC2012) (European Environment Agency 2012). However, it should be noted that the distance traveled by some individual white-tailed eagles from the center of the home range can be greater (Nadjafzadeh et al. 2016b).

Because the CLC2012 model is restricted to the European Union, it was not possible to obtain complete data for 4 locations on the border with Belarus and Ukraine; assuming 20-km buffers for the Białowieża, Gołdap, Kryłów, and Włodawa sample sites, it was only possible to obtain data for 49, 63, 54, and 65% of the landscape surface, respectively. Thus, to enable comparisons with locations with full landscape data coverage, the relative area (percentage) of each habitat in the Polish part of the 20-km buffer was used in all calculations (i.e., the areas were proportionally smaller in the case of the border locations). Spatial analyses (the extraction of particular landscape features and the area calculations) were performed using ArcMap software, version 10.3.1 (ArcGIS, ESRI, Redlands, California, USA). To compare the proportions of the habitat areas between the northern and southern regions, 2 × 2 *χ* ^2^ tests were performed, and to compare the element concentrations in birds from the northern and southern regions, a Mann–Whitney *U* test was applied. A total of 20 sites were analyzed; only two eagles were collected in each of two additional sites.

A *Q*–*Q* plot (the quantile expected from a normal distribution vs the observed quantile plot for residuals), as well as Levene’s test were used to assess whether the data met the assumptions of the linear model. Data for Ca, Hg, Zn were normalized using Box–Cox transformations, and the data for Fe were analyzed by PERMANOVA. Correlation strength was determined following Hinkle et al. (2003) as a strong correlation (*r* = |0.90–1.00|), a high correlation (*r* = |0.70–0.90|), or a moderate correlation (*r* = |0.50–0.70|). Statistical analyses were conducted in STATISTICA 12.0 (StatSoft, Inc. 2014) and PAST 3.0 (Hammer et al. 2001).

## Results

### Concentrations and possible sources of elements

The hepatic trace element concentrations in the 22 studied white-tailed eagles were ordered as follows: S > K > Na > Fe > Mg > Ca > Zn > Cu > Mn > Se > Pb > Hg > Cd > Cr > Sr > V > Sc.

Among the studied individuals, 8 (36.4%) had Pb concentrations exceeding the background level (i.e., >6.0 mg kg^−1^ dw), and 7 (31.8%) showed acute poisoning (>30.0 mg kg^−1^ dw), with very high values in 2 individuals (180.3 and 188.6 mg kg^−1^) (Table 3). Generally, 8 (53.3%) adults and none of the immatures had Pb levels exceeding the background, and 7 (46.7%) adults had Pb levels exceeding the criterion for acute poisoning. A hepatic Pb concentration of 5.54 mg g^−1^ dw was found in a single immature bird.

**Table 3:** Background and toxic concentrations (mg kg^−1^ dw) of lead, cadmium, and mercury in avian livers [reference values according to Martin et al. 2008 (A), Guitart et al. 1994 (B), Scheuhammer 1987 (C), and (D) Shore et al. (2011)] and their frequencies in livers of the studied white-tailed eagles wintering in Eastern Poland

Element	Concentration level (mg kg^−1^ dw)			
	Background	Subclinical toxicity	Moderate clinical poisoning	Acute clinical poisoning
Pb				
Reference values	0–6 (A)	6–20 (B)	20–30 (B)	>30 (A,B)
Frequency in livers of white-tailed eagles	63.6%	4.6%	–	31.8%
Cd				
Reference values	0–3.0 (C)	>3.0 (C)	–	–
Frequency in livers of white-tailed eagles	100.0%	–	–	–
Hg				
Reference values	0–6.7 (D)	> 6.7 (D)	–	>67.0 (D)
Frequency in livers of white-tailed eagles	100.0%	–	–	–

In the case of Cd and Hg, the hepatic levels found in the studied individuals were within the background concentration range (Table 3). Two immatures showed 5.19 and 5.45 mg g^−1^ dw of hepatic Hg, which were the highest Hg concentrations observed in this study. High positive relationships were found between Pb and Fe (Spearman rank correlation, *r*_s_ = 0.81), Mg and Cr (*r*_s_ = 0.81), Fe and Zn (*r*_s_ = 0.80), Fe and Cd (*r*_s_ = 0.79), Cd and Zn (*r*_s_ = 0.75), K and Mg (*r*_s_ = 0.75), K and Fe (*r*_s_ = 0.74), Ca and Sr (*r*_s_ = 0.72), and Cr and Zn (*r*_s_ = 0.71) (Table 4).

**Table 4:** Spearman correlation coefficients (*r*_s_) for all elements detected in white-tailed eagles. Strengths of significant correlations (*P* < 0.05): bolded—high correlation (*r*_s_ = |0.71–0.90|), underlined—moderate correlation (*r*_s_ = |0.51–0.70|), and italicized—low correlation (*r*_s_ = |0.31–0.50|) (according to Hinkle et al. 2003)

Element	K	S	Na	Ca	Sc	Cd	Cr	Cu	Fe	Hg	Mg	Mn	Pb	Se	Sr	V	Zn
K	1.00	0.51	0.25	−0.04	0.00	0.70	0.55	−0.08	**0.74**	0.05	**0.75**	0.58	0.60	−0.69	0.31	0.40	0.62
S	0.51	1.00	0.39	0.16	0.25	0.18	0.16	0.38	*0.43*	0.37	0.56	0.57	*0.46*	−0.65	0.21	0.38	0.28
Na	0.25	0.39	1.00	−0.53	0.24	0.01	−0.02	0.37	0.39	0.04	0.26	0.31	0.40	−0.12	−0.29	0.38	0.10
Ca	−0.04	0.16	−0.53	1.00	−0.25	0.34	0.07	−0.49	0.03	−0.06	−0.08	−0.32	0.08	−0.46	**0.72**	0.15	0.26
Sc	0.00	0.25	0.24	−0.25	1.00	−0.14	0.03	0.24	−0.08	0.13	0.08	*0.43*	−0.11	0.06	−0.25	−0.19	−0.21
Cd	0.70	0.18	0.01	0.34	−0.14	1.00	0.58	−0.52	**0.79**	0.01	0.56	0.26	0.64	−0.62	0.66	0.27	**0.75**
Cr	0.55	0.16	−0.02	0.07	0.03	0.58	1.00	0.13	0.60	0.01	**0.81**	0.34	0.41	−0.37	0.58	0.09	**0.71**
Cu	−0.08	0.38	0.37	−*0.49*	0.24	−0.52	0.13	1.00	−0.05	0.27	0.29	0.37	−0.13	0.19	−0.32	−0.11	−0.07
Fe	**0.74**	0.43	0.39	0.03	−0.08	**0.79**	0.60	−0.05	1.00	0.14	0.69	0.33	**0.81**	−0.58	*0.45*	0.29	**0.80**
Hg	0.05	0.37	0.04	−0.06	0.13	0.01	0.01	0.27	0.14	1.00	0.19	0.41	0.46	−0.06	−0.02	−0.47	−0.21
Mg	**0.75**	0.56	0.26	−0.08	0.08	0.56	**0.81**	0.29	0.69	0.19	1.00	0.66	0.56	−0.54	*0.43*	0.29	0.64
Mn	0.58	0.57	0.31	−0.32	*0.43*	0.26	0.34	0.37	0.33	0.41	0.66	1.00	0.33	−0.41	−0.10	0.05	0.12
Pb	0.60	*0.46*	0.40	0.08	−0.11	0.64	0.41	−0.13	**0.81**	*0.46*	0.56	0.33	1.00	−0.64	0.30	0.18	*0.42*
Se	−0.69	−0.65	−0.12	−*0.46*	0.06	−0.62	−0.37	0.19	−0.58	−0.06	−0.54	−0.41	−0.64	1.00	−*0.46*	−*0.48*	−0.52
Sr	0.31	0.21	−0.29	**0.72**	−0.25	0.66	0.58	−0.32	*0.45*	−0.02	*0.43*	−0.10	0.30	−*0.46*	1.00	0.21	0.70
V	0.40	0.38	0.38	0.15	−0.19	0.27	0.09	−0.11	0.29	−*0.47*	0.29	0.05	0.18	−*0.48*	0.21	1.00	*0.45*
Zn	0.62	0.28	0.10	0.26	−0.21	**0.75**	**0.71**	−0.07	**0.80**	−0.21	0.64	0.12	0.42	−0.52	0.70	*0.45*	1.00

**Fig. 2:**
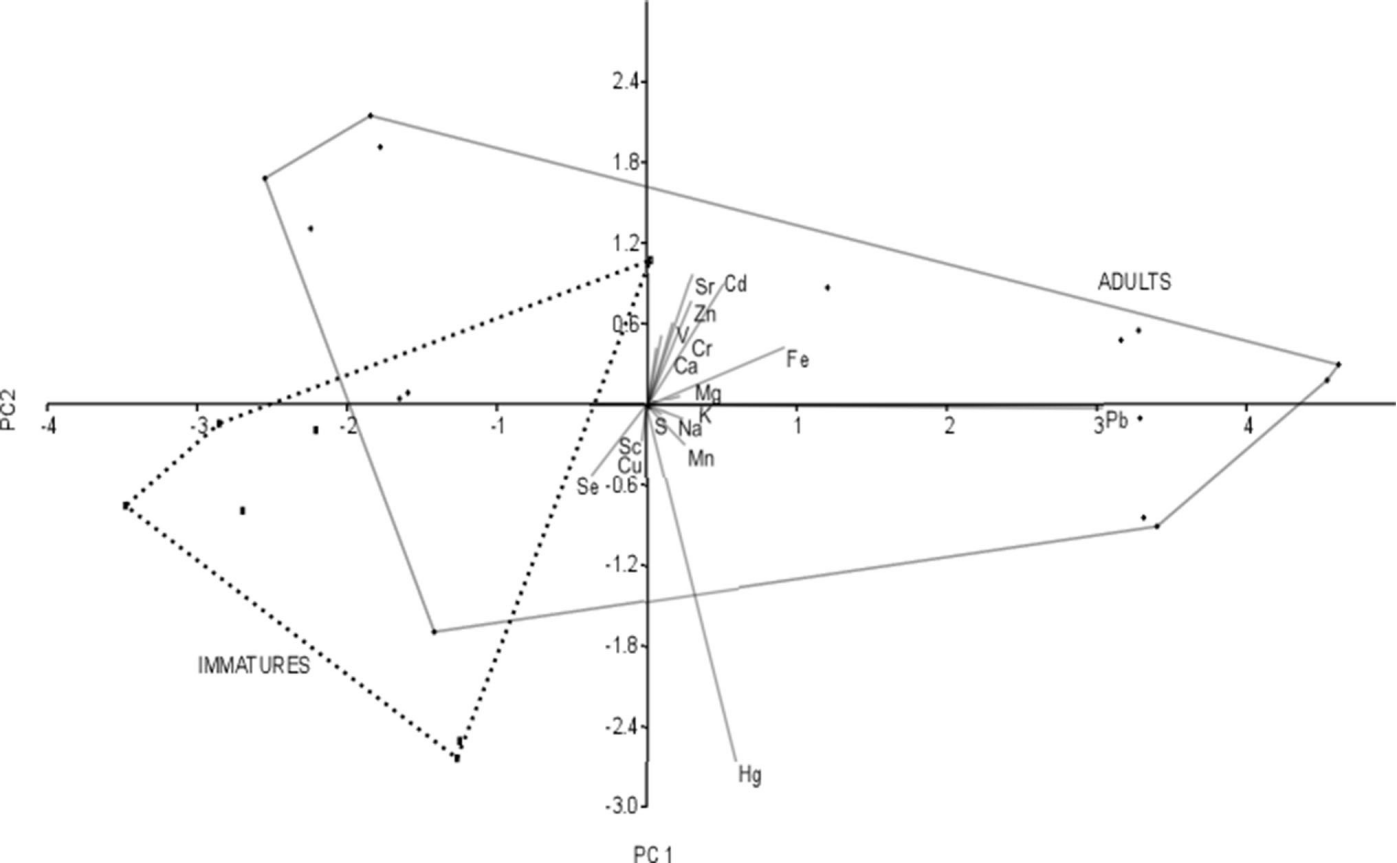
PCA biplot of element concentrations in the livers of immature and adult white-tailed eagles wintering in Eastern Poland. Convex hulls contain all samples representing a particular age category. Samples collected from adults and immatures are presented as points, and elements are presented as vectors

Principal component analysis revealed that 84.4% of the total variance was explained by the two first axes (Table 3). Samples from the adults generally clustered in different positions than those from the immatures (Fig. 2). PC1 explained 69.3% of the total variance and showed a strong positive correlation with Pb (*r* = 0.91). PC2 explained 15.0% of the total variance and was highly negatively correlated with Hg (*r* = −0.79) (Table 5).

**Table 5:** Values of the principal component loadings for the elements (ln transformed) in the studied white-tailed eagles; strongly and highly correlated values (*r* > |0.70|)

Element	Axis 1	Axis 2
K	0.07	−0.03
S	0.03	−0.02
Na	0.01	−0.01
Ca	0.02	0.13
Sc	−0.01	−0.08
Cd	0.15	0.27
Cr	0.03	0.15
Cu	0.00	−0.18
Fe	0.27	0.13
Hg	0.18	−**0.79**
Mg	0.07	0.02
Mn	0.08	−0.09
Pb	**0.91**	−0.01
Se	−0.11	−0.16
Sr	0.09	0.29
V	0.05	0.18
Zn	0.09	0.23
Eigen values	7.5	1.6
Total variance explained (%)	69.3	15.0

The majority of the immatures were characterized by low values of PC1 and PC2, i.e., by high concentrations of Hg and low concentrations of Pb. The largest group of adults (clustered in the right-hand part of the graph) was characterized by high PC1 values, reflecting high Pb concentrations. A second group of adults (clustered in the left-hand part of the graph) was characterized by low Pb values.

The Hg/Se molar ratio was calculated for all the examined liver samples and amounted to ≤0.2 in 68% of the livers. The geometric mean calculated over all specimens was 0.12 (range: 0.01–0.64).

### Intergroup differences

The concentrations of all studied elements were significantly affected by age (PERMANOVA on ln-transformed data; Bray–Curtis similarity: *F*_1,21_ = 3.53, *P* = 0.006). No significant influence of sex or an age x sex interaction on element concentrations (PERMANOVA, all *P* > 0.72) was found. The SIMPER analysis (Bray–Curtis similarity) showed that Pb, Hg, and Cd contributed the most (24, 11, and 9%, respectively) to the pattern of dissimilarity between age groups.

Univariate analyses performed separately for particular elements revealed significant age effect (ANOVA/PERMANOVA, all *P* < 0.05) for 7 elements (Ca, Cd, Cu, Fe, Pb, Se, and Zn) (Table 6). Neither sex nor age x sex interaction affected hepatic concentration of those elements significantly (ANOVA/PERMANOVA, all *P* > 0.05). Hepatic concentrations of Cr, Hg, K, Mg, Mn, Na, S, Sc, Sr, and V were not affected significantly by age, sex, or age x sex interactions (ANOVA, all *P* > 0.05).

**Table 6:** Concentrations (mg kg^−1^ dw) of the studied elements in adult and immature white-tailed eagles. Age differences detected by ANOVA or PERMANOVA(^p^) at the following *P* levels (Fe): *P < 0.05, **P < 0.01, and ***P ≤ 0.001

Element	Immatures (*N* = 7)				Adults (*N* = 15)			
	Mean	SD	Min	Max	Mean	SD	Min	Max
Ca	253.4*	125.9	132.0	471.3	397.6*	275.2	224.6	1075.9
Cd	0.16***	0.04	0.13	0.24	0.53***	0.14	0.19	0.70
Cr	0.27	0.06	0.16	0.32	0.44	0.28	0.22	1.20
Cu	23.0***	6.6	13.1	31.8	13.1***	4.9	6.3	20.0
Fe^p^	1466.5**	175.4	1325.3	1846.0	4920.5**	3534.4	1118.0	11 841.2
Hg	2.2	2.2	0.3	5.5	1.6	1.5	0.1	4.7
K	8232.1	637.7	7278.2	9052.7	9612.2	2827.8	2419.9	12 550.0
Mg	625.6	63.8	544.1	706.1	754.5	199.8	502.6	1019.7
Mn	9.6	2.8	5.1	12.8	11.4	4.8	6.0	18.5
Na	3874.3	225.4	3516.7	4139.6	3871.1	351.1	3462.3	4613.6
Pb	1.3**	1.9	0.1	5.5	48.7**	63.6	0.4	188.6
S	9234.7	885.3	7886.0	10 023.0	8712.4	1395.0	7116.7	10 960.0
Sc	0.01	0.00	0.01	0.02	0.01	0.01	0.01	0.03
Se	5.2**	2.6	2.6	9.1	3.2**	1.1	1.6	5.2
Sr	0.20	0.10	0.07	0.32	0.37	0.21	0.11	0.69
V	0.20	0.11	0.07	0.33	0.18	0.072	0.08	0.32
Zn	116.3*	34.4	69.7	159.4	183.0*	73.0	71.3	301.5

Adults had higher hepatic concentrations of Ca (ANOVA on Box–Cox-transformed data: *F*_1,21_ = 4.45, *P* = 0.049), Cd (ANOVA: *F*_1,21_ = 62.0, *P* < 0.0001), Fe (one-way PERMANOVA: *F* = 8.90, *P* = 0.009), Pb (ANOVA on Box–Cox transformed data: *F*_1,21_ = 6.38, *P* = 0.02), and Zn (ANOVA on Box–Cox transformed data: *F*_1,21_ = 4.48, *P* = 0.048) than immatures (Table 5).

Immatures had higher hepatic concentrations of Cu (ANOVA: *F*_1,18_ = 14.6, *P* = 0.001), and Se (ANOVA: *F*_1,21_ = 5.87, *P* = 0.03) than adults (Table 5).

### Regional differences in element concentrations

Inter-regional differences in the hepatic concentrations of some elements in white-tailed eagles were found. Higher concentrations of K, S, Cd, Fe, Mg, Sr, and Zn were found in the northern region, and higher concentrations of Se were found in the southern region (Mann–Whitney *U* test: *P* < 0.05; Table 7). There was a tendency (*P* = 0.06) for individuals from the northern region to accumulate higher Pb concentrations, but the hepatic levels of Na, Ca, Cr, Cu, Hg, Mn, Sc, and V in eagles from the northern and southern regions were similar (Table 7).

**Table 7:** Concentrations (mg kg^−1^ dw) of the studied elements in adult and immature white-tailed eagles combined in the northern (N) and southern (S) parts of the study area. Mann–Whitney test for inter-regional differences. Significant differences (*P* < 0.05) are bolded

Element	Region N (*N* = 14)			Region S (*N* = 8)			Mann–Whitney test	
	Median	Min	Max	Median	Min	Max	*U*	*P*
K	10 291.4	2419.9	12 550.0	7914.9	6485.8	10 216.7	22.0	**0.022**
S	9625.8	7219.3	10 960.0	7907.5	7116.7	9793.3	26.0	**0.044**
Na	3833.5	3545.3	4406.0	3761.8	3462.3	4613.6	46.0	0.517
Ca	288.8	178.1	1017.7	243.1	132.0	1075.9	37.0	0.207
Sc	0.0	0.0	0.0	0.0	0.0	0.0	52.5	0.779
Cd	0.5	0.2	0.7	0.2	0.1	0.5	16.5	**0.008**
Cr	0.4	0.2	1.0	0.3	0.2	1.2	32.5	0.116
Cu	15.3	6.6	28.2	17.8	6.3	31.8	44.0	0.433
Fe	5003.2	1118.0	11 841.2	1466.5	1175.3	3049.0	22.0	**0.022**
Hg	0.9	0.1	4.7	1.4	0.2	5.5	49.5	0.682
Mg	766.0	533.4	1019.7	564.1	502.6	743.6	21.0	**0.019**
Pb	36.9	0.1	188.6	0.7	0.2	1.4	28.0	0.061
Mn	10.2	5.1	18.5	8.2	6.0	12.6	38.0	0.232
Se	2.9	1.6	4.6	4.4	3.0	9.1	15.0	**0.006**
Sr	0.3	0.1	0.7	0.2	0.1	0.7	38.0	**0.034**
V	0.2	0.1	0.3	0.2	0.1	0.3	24.5	0.231
Zn	170.2	72.0	301.5	95.1	69.7	252.8	24.0	**0.032**

The highest Hg concentrations were found in immatures from locations in the southern region situated close to the Bug River in Kryłów and Włodawa. The highest hepatic concentrations of Pb were recorded in adults collected in the Masurian Lake District (central part of the northern region). A group of adults from the left-hand part of the graph representing low Pb values originated from the southern region and the western part of the northern region.

### Relationships between element concentrations and landscape features

For all individuals combined (*N* = 22), a significant positive relationship was found between the relative area of wetlands and the Pb concentration (Spearman correlation coefficient: *r*_s_ = 0.45, *P* = 0.04), and there was also a significant positive relationship between the relative area of water bodies and the Cd concentration (*r*_s_ = 0.44, *P* = 0.04). A series of significant negative correlations was found between the relative area of artificial surfaces and K (*r*_s_ = −0.44, *P* = 0.04), S (*r*_s_ = −0.57, *P* = 0.005), and Pb (*r*_s_ = −0.59, *P* = 0.004), and there was one positive correlation with Se (*r*_s_ = 0.66, *P* = 0.0009).

No significant relationships were found between the concentration of any element and the relative area of forest (Spearman correlations, all *P* > 0.22), pasture (all *P* > 0.11), or arable land (all *P* > 0.41).

## Discussion

To our knowledge, this is the first study to investigate the possible sources of trace elements in the livers of white-tailed eagles in Central Europe and one of the few studies to investigate the concentrations of elements in this species in relation to sex and age. This study presents element contamination levels in winter in a habitat mosaic of forest, lakes, and extensive agriculture that is characteristic of Eastern Poland.

PCA and SIMPER analyses revealed the two main elements that differed the most in the livers of the studied white-tailed eagles: Pb and Hg; most individuals with a high level of one of these elements also had a low value for the other, which suggests different sources of origin for both elements (Kalisinska et al. 2014a). Although fertilizers are often blamed for being a source of Hg contamination (Mortvedt 1995; Otero et al. 2005) and coal combustion is cited as a significant source of Hg emissions (Pacyna et al. 2006; Hlawiczka and Cenowski 2013; Zajusz-Zubek and Konieczyński 2014) in Poland, the main source of Hg contamination in this study was aquatic prey (fish and birds). This was previously reported for white-tailed eagles from Western Poland (Falandysz et al. 2001) and can be explained by the transport of highly contaminated water in the Bug River northwards from the Ukrainian Lviv–Volyn Coal Basin (Wolska et al. 2008; Skorbiłowicz 2014).

The significant correlations of the hepatic Pb concentration with the relative area of wetlands (positive) and artificial surfaces (negative) indicate a non-industrial aquatic origin of this element in the white-tailed eagle. In the areas of Eastern Poland with ice-covered lakes in winter, white-tailed eagles mainly forage on the carrion of ungulates and dead mute swans and mallards (Zawadzka 1999; Zawadzka et al. 2006), which may be contaminated by lead from shot hunting ammunition (Perrins et al. 2003; Mateo 2009) and corresponds with the hepatic Pb levels in eagles from the Masurian Lake District.

### Age and sex differences in element concentration levels

Significant age-related differences in the hepatic concentrations of some elements were found in adult eagles, which exhibited higher levels of Ca, Cd, Fe, Pb, and Zn and lower concentrations of Cu and Se compared with immatures. Similar age-related differences in renal and hepatic Cd concentrations have been found in another raptor, the common buzzard (Carneiro et al. 2014), and lower hepatic concentrations of Cd, Pb, Cu, and Fe have also been reported in immature common kestrels from South Korea (Kim and Oh 2015). However, in some studies, including of *Haliaeetus* sp. eagles, no age-related differences have been found; no significant age-related differences in Cd, Cu, Fe, Hg, Mg, Mn, Mo, Se, and Zn concentrations have been found in the livers of the American counterpart to the white-tailed eagle, the bald eagle, *Haliaeetus leucocephalus*, from Alaska (Stout and Trust 2002). Similar hepatic Pb levels have been reported in immature and adult white-tailed eagles from Sweden (Helander et al. 2009). Therefore, the differences in the contamination levels between adults and immatures observed in our study may be explained by age-related differences in wintering strategies and hunting skills. Sedentary adults wintering in their territories forage in well-known feeding areas. By contrast, migrating and less-experienced immatures hunt in new areas (Cramp and Simmons 1980; Mizera 2004; Nadjafzadeh et al. 2016a). Moreover, experienced adults are more successful when hunting for agile prey, such as waterfowl (Nadjafzadeh et al. 2013; Nadjafzadeh et al. 2016a); many such game birds are often contaminated with Pb.

Similar to other studies on raptors (e.g., Castro et al. 2011; Bedrosian et al. 2012), this study did not find significant sex-related differences in hepatic element concentrations. Studies of German and Austrian white-tailed eagles revealed no sex-related differences in the hepatic concentrations of Cd, Hg, and Pb (Kenntner et al. 2001), and similar hepatic levels of Pb regardless of sex have been reported in Sweden (Helander et al. 2009). Additionally, sex-related differences in Pb hepatic concentrations have been reported to be rare in other raptors (Franson et al. 1983; Wayland et al. 1999; Castro et al. 2011; Carneiro et al. 2014). The lack of sex-related differences in white-tailed eagles may be explained by the only slight reverse sexual dimorphism (RSD), which does not result in differences in foraging ecology. Sex-related differences in prey preferences have been documented in raptor species with more pronounced RSD (e.g., Newton 1979; Sunde et al. 2003; Bujoczek and Ciach 2009), which allows for foraging niches to be segregated to avoid competition (Temeles 1986; Sunde et al. 2003).

### Concentrations of selected elements

#### Sulfur

The liver serves as a target for the deposition of many substances that are important for the organism (including macro- and microelements), and it is also the organ involved in detoxification processes (the elimination of toxic elements such as Cd, Hg, and Pb). A major chemical property of many toxic metals, including Cd, Hg, and Pb, is their capacity to strongly bind to thiol residues, which are used by most organisms for detoxification; therefore, heavy metal detoxification by glutathione necessitates high amounts of sulfur (Tamas and Martinoia 2006). Furthermore, the detoxification of metals based on metallothionein, a cysteine-rich protein, is also sulfur-dependent. To cope with this vital sulfur requirement, sulfur metabolism is normally directed to the intake of methionine and cysteine for protein synthesis, but neither cysteine nor methionine is stored in the body. Any dietary excess is readily oxidized to sulfate, excreted in the urine (or reabsorbed depending on dietary levels) or stored in the form of glutathione (Nimni et al. 2007), and these processes are related to the supply of sulfur, which is collected in the form of amino acids, to a healthy body (Nimni et al. 2007, Netto et al. 2014, Toohey 2014). The birds examined in this study accumulated 40–50% less S in their livers (Table 6) than eagles from Western Poland (Falandysz et al. 2001) (mean: 17 000 mg kg^−1^ dw), which may indicate (in the context of very high levels of hepatic Pb) problems in the supply of sulfur necessary for detoxification.

#### Lead

The high Pb concentration levels reported in the presented study (up to 180 mg kg^−1^) may be explained in terms of the high contribution of the carrion of shot game mammals and waterfowl to the winter diet of white-tailed eagles; when fish availability sharply declines, the birds switch to foraging on waterfowl and carrion (van Rijn et al. 2010; Nadjafzadeh et al. 2013). Thus, the highest frequencies of Pb poisoning in eagles are reported in autumn and winter (Helander et al. 2009; Nadjafzadeh et al. 2013). The Pb concentrations in individuals collected in the northern part of the study area tended to be higher than those in the southern part. The northern region, the NE Poland, serves as the wintering grounds and stop-over site for many waterfowl species (Zawadzka 1999; Zawadzka et al. 2006).The eagles’ habit of preying on ducks killed with lead ammunition may explain the elevated Pb levels in white-tailed eagles from this area. Elevated levels of Pb in the study area are a consequence of the lack of restrictions on the use of lead hunting ammunition in Poland (in contrast to many other European countries; Mateo 2009). Reports from different countries suggest that the remains of hunted big game mammals may be a significant source of dietary lead exposure for large eagles in areas with low waterfowl availability (Helander et al. 2009; Bedrosian et al. 2012). In the USA, the frequency of lead ingestion in both golden eagles, *Aquila chrysaetos*, and bald eagles did not drop after lead shot was banned for waterfowl hunting (Kramer and Redig 1997), which indicates mammal carcasses as an alternative source of Pb exposure. Moreover, areas and periods with a high incidence of lead poisoning in eagles were not correlated with waterfowl hunting in both the western USA and the Great Plains (Miller et al. 1998; Wayland et al. 2003). In our study, the frequency (32%) of birds with hepatic Pb levels indicating poisoning (i.e., >20 mg kg^−1^ dw) is higher than that reported in Germany (24%; Kenntner et al. 2001) or Sweden (12.5%; Helander et al. 2009), but similar to the frequency reported for bald eagles from Michigan and Minnesota in the USA (30%; Nam et al. 2012). Moreover, the frequency of birds with background hepatic Pb concentrations, i.e., <6.0 mg kg^−1^, was lower in our study (64%) compared with Sweden (85%; Helander et al. 2009). Severe Pb intoxication can cause serious disorders, such as depression, paralysis, weakness of the legs or wings, and neuropathological damage in the brain (encephalopathy), which is manifested as an impairment of the blood–brain barrier, as well as enlargement of the gall bladder (Helander et al. 2009; Stauber et al. 2010; Nam et al. 2012). Thus, Pb contamination may considerably affect the eagle mortality rate.

#### Cadmium

The liver is one of the two main organs subjected to Cd accumulation (Garcia-Fernandez et al. 1996; Nam et al. 2005a, b; Wayland and Scheuhammer 2011); the mean hepatic Cd concentration (mg kg^−1^ dw) found in this study (0.35) was approximately 9 times lower than that indicative of increased environmental exposure (Burgat 1990; Battaglia et al. 2005). By contrast, lower arithmetic mean Cd concentrations (0.15) have been reported for white-tailed eagles from Northwestern Poland (Falandysz et al. 2001). The positive relationship between the relative area of water bodies and hepatic Cd concentrations reported in our study may be explained in terms of waterfowl hunting in lakeland areas. The Cd concentrations in the kidneys of most wild freshwater ducks, which are the primary prey of the studied species, are relatively high, i.e., 2–8 (Scheuhammer 1987; Binkowski et al. 2013), and Cd bioaccumulation is accompanied by changes in the levels of some essential elements, including Zn (Brzoska and Moniuszko-Jakoniuk 2001), which was reflected in our study in the significantly high correlation between both elements (*r*_s_ = 0.75). Positive correlations between tissue concentrations of Cd with Zn have been reported for many species of birds (Furness 1996; Nam and Lee 2006; Kim et al. 2008, 2009). Increased liver concentrations of Zn (Chmielnicka et al. 1989; Lopez-Alonso et al. 2002) decrease the availability of this element for other tissues (for example, bone) and many biochemical processes (Brzoska and Moniuszko-Jakoniuk 2001), but an increased Zn supply may reduce Cd absorption and accumulation, thus preventing or reducing the adverse actions of Cd. However, Zn deficiency can intensify Cd accumulation and toxicity (Brzoska and Moniuszko-Jakoniuk 2001). Studies of mammals have revealed that Se can enhance Cd accumulation in mammal tissues and prevent acute Cd toxicity (Wahba et al. 1993), which corresponds with our results showing a negative correlation between Cd and Se in the livers of the examined white-tailed eagles.

#### Mercury

Mean hepatic concentrations (mg kg^−1^ dw) of Hg, both in our study (1.046) and in a study from the South Baltic (mean 1.91; Kalisinska et al. 2014a), confirm a current decrease in environmental Hg concentrations, perhaps due to various activities aimed at limiting its industrial emissions. At the beginning of the 1990s, white-tailed eagles from Northwestern Poland accumulated an average of 5.8 (range: 0.6–21.0) of hepatic Hg (Falandysz et al. 2001). Bald eagles from British Columbia (Canada) have been shown to accumulate ~10 times more Hg (mean: 11.8, range: 0.5–17.2) than the birds in our study (Weech et al. 2003). The accumulation of Hg is strongly determined by local conditions. Thus, the level of contamination observed in the studied birds from Eastern Poland, which has a low level of industrialization, was relatively low (<5.45).

The main source of Hg for the examined white-tailed eagles was aquatic prey, including fish and birds, as indicated by the possible accumulation of this element in their internal organs. Łuczyńska (2005) reported that the total mercury content (mg kg^−1^ dw) ranged from 0.076 to 0.902 in the muscle of pike, from 0.074 to 0.278 in roach, and from 0.104 to 1.277 in perch, *Perca fluviatilis*. Kalisinska et al. (2013) determined the Hg concentrations in the following organs in mallard, a duck that is frequently captured by white-tailed eagles: breast muscle (median: 0.114, range: 0.008–0.938), liver (median: 0.203, range: 0.016–0.966), and kidney (median: 0.270, range: 0.010–1.499).

Mercury speciation was not performed in the present study; therefore, we only had information concerning the total amount of Hg (THg). However, this was sufficient to estimate the threat from this element to the studied eagles. All forms of mercury are toxic to birds and mammals, but one should pay particular attention to the toxicity of methylmercury (MeHg) due to its high bioavailability and neurotoxicity (Nam et al. 2012). Inorganic Hg (InHg) that has been subjected to bio-transformation by microorganisms in sediments is the main source of MeHg (Wiener et al. 2003). Considering the data obtained in this study, it should be noted that MeHg occurs at a high ratio in fish muscles (>80% of THg) (Kalisinska et al. 2017); methylmercury demethylation occurs in the liver and kidneys, leading to the less toxic InHg, which can be excreted (Wiener et al. 2003).

#### Selenium

Poland belongs to a group of countries for which Se deficiency in the environment has been reported (Nowakowska et al. 2014; Kalisinska et al. 2014b, c). This study showed that higher hepatic Se levels were found in white-tailed eagles from Southeastern Poland (see Fig. 1) than in those from Northeastern Poland (Table 7). There was a positive relationship between the hepatic Se level and the urbanized area around the sampling sites. This may indicate that coal combustion is a significant selenium source, which has also been reported previously (Hartzell 2014). Especially in Southeastern Poland, the consumption of coal for heating purposes is very high; hard coal fulfills 3/4 of the heating demand in households in the studied region of Lublin (Szul 2011) in contrast to Northeastern Poland, where more frequently wood is used.

Two forms of Hg, inorganic Hg (InHg) and methylmercury Hg (MeHg), can inhibit the selenoenzyme glutathione peroxidase, which is crucial for antioxidant regulation (Branco et al. 2012). Therefore, it is important to examine the total Hg (THg) concentration, as well as the ratio of THg to total Se; studies of bird livers have shown positive correlations between concentrations of the particular forms of Hg and total Hg (THg), as well as between the concentrations of Se and THg (Scheuhammer et al. 2008; Kalisinska et al. 2014b, c). The molar ratios of THg to Se obtained in this study correspond to the data obtained for inland and wetland birds, as well as seabirds, for which the reported Hg:Se molar ratio ranged between 0.1 and 0.2 (Kim et al. 1996; Petrie et al. 2007; Conover and Vest 2009).

#### Iron

In the studied white-tailed eagles, a relatively high concentration (mg kg^−1^ dw) of Fe (mean 2753) was detected compared with data from Northwestern Poland [mean 1200 (Falandysz et al. 2001); 1726 (Kalisińska et al. 2006)]; similar values (2287) were detected in the livers of common buzzards, *Buteo buteo*, from the Netherlands (Jager et al. 1996). The hyper-accumulation of Fe in the studied white-tailed eagles may be explained as a response to Pb poisoning (Ochiai et al. 1992; Lewis et al. 2001; Kalisinska et al. 2008a). A similar mechanism has also been reported for bats, in which a high level of blood Pb was accompanied by a high accumulation of Fe in the liver (Farina et al. 2005); this phenomenon was reflected in this study by a high correlation between both elements (*r*_s_ = 0.81). On the other hand, the high accumulation of Fe may also be indicative of strong bacterial and helminthological infections (Kalisinska et al. 2008a). According to Harrison’s rule, large avian species such as white-tailed eagles can host richer parasite communities (Poulin 1998), and white-tailed eagles are hosts of a broad spectrum of endoparasites (Krone et al. 2004, 2006, 2008; Kalisinska et al. 2008b). We do not know the infection status of the studied individuals, but dissection revealed that two individuals with elevated levels (mg kg^−1^ dw) of both Fe (4136.5 and 5782.3, respectively) and Pb (9.78 and 63.56, respectively) had fungal infections in their internal organs.

#### Copper

Hepatic accumulation (mg kg^−1^ dw) of Cu in the present study (mean: 14.69) was comparable to results for white-tailed eagles from Northwestern Poland (13.0 and 16.08; Falandysz et al. 2001; Kalisińska et al. 2006). Elevated hepatic concentrations of Cu are related to the depletion of fat and protein resources (Esselink et al. 1995; Jager et al. 1996), as Cu mainly originates from ingested aquatic prey, such as fish and waterbirds. The most important avian prey components of white-tailed eagles in Poland, such as coots and mallards (Zawadzka 1999; Zawadzka et al. 2006), accumulate 16.1 and 39.5–127.0 of hepatic Cu, respectively (Nam et al. 2005a; Kim and Oh 2012; Binkowski et al. 2013). Even higher levels of Cu (>500 mg kg^−1^ dw) have been reported in the livers of supplementary white-tailed eagle prey, including the grey heron, *Ardea cinerea*, and the mute swan, *Cygnus color* (Horai et al. 2007; Schummer et al. 2011; Komosa et al. 2012), and fish may also serve as an important source of Cu. High accumulation of Cu and Mn has been reported in the muscle and livers of fish from Eastern Poland (bream, northern pike), ranging from 6.3 to 167.0 mg kg^−1^ dw (Radwan et al. 1990).

## Conclusions

In this study, we assessed the hepatic levels of trace elements in white-tailed eagles wintering in relatively unpolluted Northeastern Poland, and we found age-related differences in some element concentrations that may be explained by differences in wintering strategies and hunting skills. Our study confirmed that the internal organs of large raptors, such as the white-tailed eagle, are good bioindicators of environmental contamination. Lead ammunition continues to pose a serious threat to the health and lives of white-tailed eagles in Poland (32% of the studied individuals had acute lead poisoning). Our results and documented toxic risk for wildlife and human from exposure to lead from ingested shot ammunition (e.g., Knott et al. 2010) indicate a serious need to ban the use of lead hunting ammunition in those parts of Europe (including Poland) where it is still allowed. The regulatory actions adopted by European countries are diverse, resulting from lack of regulations (e.g., Poland) through ban on lead shot for hunting in wetlands, and/or for the hunting of waterbirds to ban to all hunted species and areas (Mateo 2009). The United Nations Convention on Migratory Species, has called to phase out all lead ammunition until 2017. Unfortunately, so far, use of lead ammunition use in the European Union is regulated only on a limited basis (banned in wetlands) under the EU chemicals regulation REACH (Registration, Evaluation, Authorization and Restriction of Chemicals).

We are aware of some limitations of our study. It was based on a non-random sample of the population, i.e., injured birds delivered to a veterinary clinic, sometimes in a sub-lethal state. Some of the studied birds might have been poisoned by food, which may have affected their hepatic element concentrations. However, the study presents current data on elemental contamination in this sentinel species of high conservation priority. The state of its health (i.e., contamination level) is important to assess the condition of its population. The results of our study are important from both an ecotoxicological and a conservation perspective.
